# Gene Dosage Experiments in Enterobacteriaceae Using Arabinose-regulated Promoters

**DOI:** 10.21769/BioProtoc.2396

**Published:** 2014-07-20

**Authors:** Sanchari Bhattacharyya, Shimon Bershtein, Eugene I Shakhnovich

**Affiliations:** 1 Department of Chemistry and Chemical Biology, Harvard University, Cambridge MA, USA; 2 Department of Life Sciences, Ben-Gurion University of the Negev, POB 653, Beer-Sheva, Israel

**Keywords:** Gene-dosage toxicity, Arabinose, Protein abundance, Western-blot, Fitness, Over-expression, Plasmid

## Abstract

This protocol is used to assay the effect of protein over-expression on fitness of *E. coli*. It is based on a plasmid expression of a protein of interest from an arabinose-regulated pBAD promoter followed by the measurement of the intracellular protein abundance by Western blot along with the measurement of growth parameters of *E. coli* cell expressing this protein.

## Background

Gene dosage experiments are crucial for understanding the effects of protein over-expression on fitness and determining the optimal levels of protein abundance. Several genes are toxic even when expressed at very low levels. It is therefore important to express the protein from a tightly regulated promoter to minimize leaky expression. Here we have elucidated conditions for expression of proteins under the well-characterized arabinose induced pBAD promoter, and designed protocols to measure the intracellular abundance and fitness of *E. coli* cells harboring the overexpression plasmid.

## Materials and Reagents

1. Sterile tips
2. 1.5 ml microfuge tubes (Corning, Axygen^®^, catalog number: MCT-175-C)
3. 14 ml polypropylene tubes for bacterial culture (Corning, Falcon^®^, catalog number: 352059)
4. 50 ml tubes for bacterial culture (Corning, Falcon^®^, catalog number: 352070)
5. Sterile needle for inoculation (Thermo Fisher Scientific, Thermo Scientific™, catalog number: 253988)
6. Honeycomb plates (Bioscreen, catalog number: 95025BIO)
7. Gene of interest cloned in pBAD/MCS-vector (https://www.embl.de/pepcore/pepcore_services/strains_vectors/vectors/bacterial_expression_vectors/popup_bacterial_expression_vectors/ obtained from European Molecular Biology Laboratory EMBL) using appropriate restriction sites in the multiple cloning site
8. *E. coli* BW27783 cells (CGSC, catalog number: 12119)
9. LB/Agar plates
10. Ampicillin (Sigma-Aldrich, catalog number: A0166)
11. Sodium phosphate buffer, pH 7.4
12. Tris-HCl pH 8.0
13. Pierce BCA protein assay kit (Thermo Fisher Scientific, Thermo Scientific™, catalog number: 23227)
14. 4× protein gel loading dye (Thermo Fisher Scientific, Invitrogen™, catalog number: NP0007)
15. Pre-cast 12% Bis-Tris SDS polyacrylamide gel (Bio-Rad Laboratories, catalog number: 3450123)
16. Trans-blot Turbo midi nitrocellulose transfer packs (Bio-Rad Laboratories, catalog number: 1704158)
17. Antibody raised against the protein of interest
18. Western-breeze Chromogenic Immunodetection kit (Thermo Fisher Scientific, Invitrogen™, catalog number: WB7105 for anti-rabbit)
19. M9 salts (BD, Difco™, catalog number: 248510)
20. Magnesium sulfate heptahydrate (MgSO_4_·7H_2_O) (Sigma-Aldrich, catalog number: 230391)
21. Thiamine (Sigma-Aldrich, catalog number: T1270)
22. Casamino acid (AMRESCO, catalog number: J851)
23. Glucose (Sigma-Aldrich, catalog number: G7021)
24. L(+) arabinose (EMD Millipore, catalog number: 178680)
25. 10× Bugbuster reagent (Novagen, catalog number: 70921-3)
26. Benzonase nuclease (EMD Millipore, catalog number: 70664)
27. Supplemented M9 medium (see Recipes)
28. Lysis buffer (see Recipes)
29. L(+) arabinose solution (Concentrations used) (see Recipes)

## Equipment

1. Pipettes
2. Incubator
3. Heated Orbital shaker
4. Centrifuge (Eppendorf, models: 5810 R and 5417 R)
5. Spectrophotometer (BioTek Instruments, model: PowerWave HT)
6. Rotator-mixer
7. Trans-Blot Turbo transfer system (Bio-Rad Laboratories, catalog number: 1704155)
8. Bioscreen C (Growth Curves USA)

## Software

1. ImageJ software

## Procedure

- For abundance measurement
  1. Transform pBAD plasmid containing the gene of interest in to electro-competent BW27783 cells (see Notes, point #1), and spread on LB/Agar plates containing 100 µg/ml ampicillin. Incubate plates overnight at 37 °C.
  2. Next day, pick a single colony from the plate and inoculate into 2 ml of supplemented M9 medium (see Recipes and Notes, point #2) containing 100 µg/ml of ampicillin. Grow overnight with shaking (250 rpm) at 37 °C in 14 ml polypropylene tubes.
  3. Next day, dilute 500 µl of the overnight culture 1/100 in to 50 ml of fresh M9 medium containing 100 µg/ml of ampicillin and varying concentrations of arabinose (0–0.05%) (see Recipes), and grow for 4 h with shaking (250 rpm) at 37 °C.
  4. After 4 h, measure OD_600_ of the cultures, and spin down in a table-top centrifuge at 3,000 × *g* for 15 min. Aspirate as much culture supernatant as possible. Store the cell pellets at −20 °C.
  5. Prepare the lysis buffer, which is the buffer of choice (50 mM sodium phosphate buffer, pH 7.4, 10 mM Tris-HCl pH 8.0, *etc.*) supplemented with the detergent Bugbuster and Benzonase nuclease (see Recipes below). Based on the measured OD_600_ of the cultures, re-suspend the pellets in the prepared lysis buffer such that the final OD_600_ is 2.0 (see Notes, point #3). Allow the lysis to proceed for 20 min at room temperature on a rotator-mixer.
  6. Following lysis, spin down the cell debris for 30 min at 7500 × *g* in a centrifuge that has been pre-chilled at 4 °C.
  7. Separate the supernatant and quantify the total protein in each sample using the BCA assay kit using the manufacturer’s instructions.
  8. Load 20 µl of the supernatants on a 12% Bis-Tris SDS polyacrylamide gel, after required dilution of the samples (see Notes, point #4).
  9. Resolve the samples at a voltage gradient of 10 V/cm of gel.
  10. Once the dye front has reached the base, the gel is ready to be transferred to a membrane for blotting. Use pre-assembled nitrocellulose membrane sandwiches (see Notes, point #5).
  11. Following transfer, wash the membrane with water two times to remove transfer buffer components and weakly bound proteins. From this step onwards, use Invitrogen’s Western-breeze Chromogenic Immunodetection kit to develop the membrane.
- For growth rate measurement
  1. From step A2 above, dilute the overnight culture to a final OD_600_ of 0.01 (see Notes, point #3) into fresh M9 medium containing 100 µg/ml of ampicillin and varying concentrations of arabinose (see Recipes). Aliquot 150 µl of this into three wells of the honeycomb Bioscreen plate (see Notes, point #6). This serves as replicates for a single colony. To obtain standard error of biological replicates, inoculate 3 independent colonies, and subject each of them to varying arabinose concentration.
  2. Aliquot 150 µl M9 medium into three independent wells. This serves as the background for OD measurements.
  3. Measure OD_600_ values at 15 min intervals over a period of 12 h in Bioscreen C system at 37 °C with shaking (see Notes, point #6).

## Data analysis

**Figure 1:**
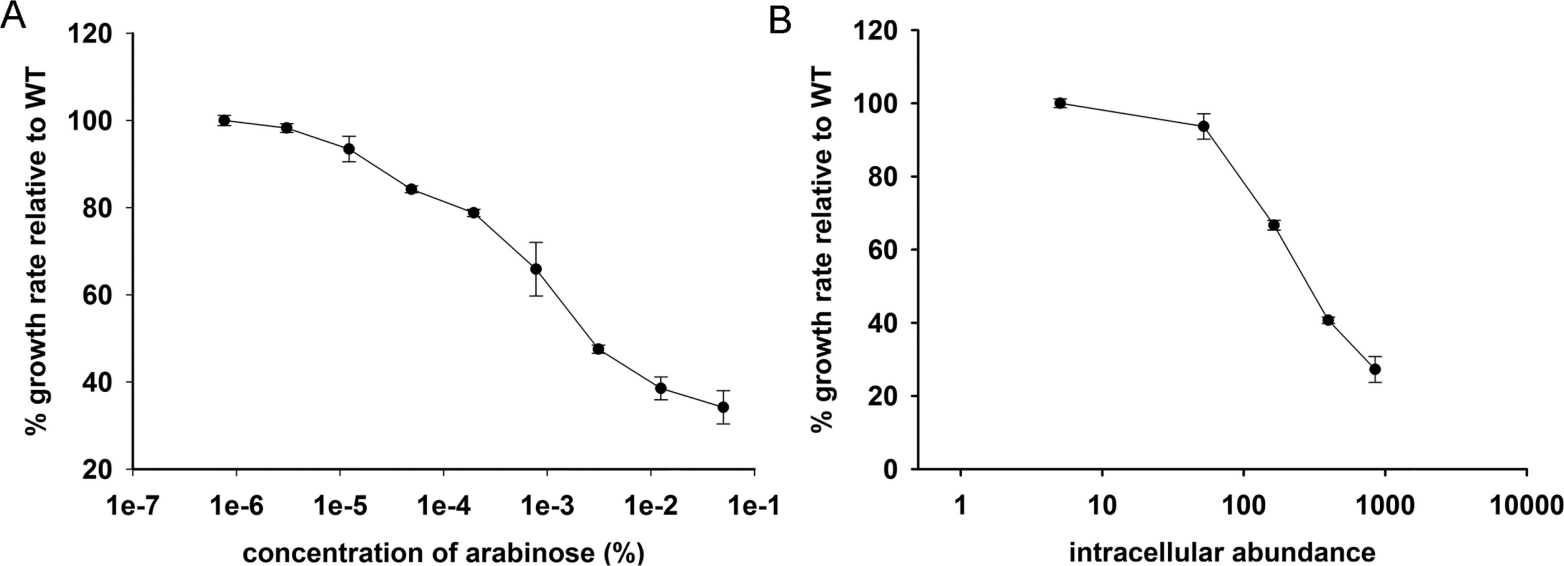
Representative gene-dosage toxicity curves for *E. coli* Dihydrofolate Reductase (DHFR) expressed from pBAD-plasmid (Bhattacharyya *et al.*, 2016) A. Plot of relative growth rate as a function of arabinose concentration; B. Plot of relative growth rate as a function of intracellular abundance of DHFR protein.

1. Use ImageJ software to evaluate the band intensities from the Western blot (For a complete tutorial, please visit ‘https://imagej.nih.gov/ij/docs/guide/146-30.html#toc-Subsection-30.13’).
2. Normalize the intensity by the total protein concentration obtained by BCA assay, and also scale up the values by the dilution factor. If the protein of interest is an endogenous *E. coli* protein, then calculate the fold-overexpression of the protein of interest based on the intensity obtained for untransformed BW27783 cells (set to 1). For a foreign protein, the expression level obtained with 0% arabinose should be set to 1.
3. Fit the growth curves obtained from Bioscreen C as *OD* vs. time (*t*) using the following 4-parameter Gompertz equation to obtain growth rate parameters (Adkar *et al.*, 2017).
  $$\text{ln}(OD)=\text{ln}(OD_{0})+\text{ln}(K)\text{exp}[-\text{exp}(-\frac{t-\lambda }{b})]$$
  Where, *K* is the fold-increase over initial population at saturation, *b* is the shape factor and defined as *b* = ln(*K*)/(*μ*·exp(1)) where *μ* is the maximum growth rate, and the lag time *λ* is the time taken to achieve the maximum growth rate.
4. Make a plot of gene-dosage effect using measured growth rates and intracellular protein abundance (Figure 1) (see Notes, point #7).

## Notes

1. The transporter araE in *E. coli* helps in uptake of arabinose from the medium. However, as the expression of araE is all or none in WT *E. coli* cells (*e.g.*, MG1655), induction with arabinose results in a heterogeneous population. BW27783 that is used in this protocol is a strain of *E. coli* MG1655 that has been engineered to constitutively express araE, resulting in a uniform and homogeneous uptake of arabinose (Khlebnikov *et al.*, 2001).
2. The gene-dosage experiments can be done in any medium of choice (LB, M9, *etc.*). However over-expression of the protein of interest may show different phenotypes in different media conditions. For example as discussed in (Bhattacharyya *et al.*, 2016), over-expression of *E. coli* Dihydrofolate Reductase (DHFR) was found to be toxic in supplemented M9 medium, but not in LB medium. We therefore suggest choosing the growth medium which allows the study of the phenotype of interest.
3. OD_600_ is defined for 1 cm path-length.
4. The typical amount of total protein from cell lysate loaded on to the gel was 5 µg. However, for higher levels of induction, dilution will be necessary. We therefore suggest doing a pilot experiment to find the necessary dilution.
5. Instead of pre-assembled nitrocellulose membrane sandwiches, one can prepare their own using pre-cut nitrocellulose membranes, blotting papers, and tris-glycine-methanol transfer buffer to perform this step successfully.
6. Bioscreen C is an absorbance based microplate reader that is used to measure growth curves of microorganisms (http://www.growthcurvesusa.com/description.html). As opposed to a conventional microplate reader, which can measure 96 wells at a time, honeycomb plates used in Bioscreen C have 100 wells, and two plates can be used at a time. The design of the honey comb plate ensures uniform temperature across the wells without any significant condensation/evaporation, thereby greatly reducing errors among replicates. However, it should be mentioned that in absence of Bioscreen C instrument, any conventional microplate reader or spectrophotometer with cuvette can also be used to perform this step successfully.
7. If it is desirable to find out the absolute amount of protein required to achieve a particular growth inhibition, it can be done by purifying the protein of interest, estimating its concentration and then using a known amount of the purified protein as a standard/reference to estimate the amount of protein from the crude lysate at each given induction level.

## Recipes

1. Supplemented M9 medium (1 L)
  11.28 g of M9 salts (Difco) (identical to the classical M9 salts recipe in Molecular Cloning by Maniatis)
  1 ml 1 M MgSO_4_
  5 µl 100 mM thiamine
  10 ml 10% casamino acid
  10 ml 20% glucose
  Make up the volume to 1,000 ml, sterilize by filtration
2. Lysis buffer (10 ml)
  1 ml 10× Bugbuster reagent
  10 µl Benzonase nuclease
  Make up the volume to 10 ml using buffer of choice
3. L(+) arabinose solution (concentrations used)
  Start with 0.05%, and then do 4-fold serial dilution up to 1.22 × 10^−5^%, in addition to 0% arabinose
